# Perceptions of media influence and performance among politicians in European democracies

**DOI:** 10.1177/17480485221146088

**Published:** 2022-12-21

**Authors:** Peter Maurer

**Affiliations:** 4209 Karlstad University, Karlstad, Sweden

**Keywords:** Media influence, perception, media performance, political elites, media and democracy, mediatisation, comparative survey

## Abstract

This study explores politicians’ subjective views of the mediatisation of politics and the implication it has for their satisfaction with democracy. Based on previous research, we hypothesise a negative effect of their perception of media influence on their evaluation of the news media’s performance as a public informant. These perceptions directly and indirectly influence politicians’ satisfaction with democracy. The relationships were tested with a Structural Equation Model (SEM) based on comparative survey data from politicians in seven democracies (Austria, Denmark, France, Germany, Spain, Sweden, Switzerland). Results show that a strong influence runs from politicians’ perception of the media’s performance as regards public information to their satisfaction with the functioning of democracy. This influence is stable across countries. The SEM thus may provide a good explanation for why some politicians attack legacy media and excessively use social media to communicate with voters. Results also point to risks of media-driven democracies.

## Introduction

In a liberal democracy, the relationship between the media – understood as a political institution (Cook, 2006) – and the political system can be conceptualised as a social contract (Strömbäck, 2005) in which the media enable voters to make informed electoral choices by relating the debate between political parties in an objective manner. The media also serve as an indispensable information and communication platform for politicians (Van Aelst and Walgrave, 2016) by – ideally – providing an open arena for political discourse. Yet, with advanced mediatisation (e.g., Asp, 2014; Kepplinger, 2007; Strömbäck and Esser, 2009), political processes have become more dependent on the media, and political actors may feel constrained in their ability to manage public support by an autonomous media system willing to set the political agenda. As Kepplinger (2007: 10) observes, ‘the framing of the issues by media reports defines the boundaries of legitimate action taken by decision makers’. The present article focuses on politicians’ subjective perceptions of this power of the media and the implications for how they perceive the media’s political role.

Research on the perceptions of politicians in some advanced democracies suggests that they recognise the power position of the media and believe that the media's narratives affect the opinions of voters (Cohen et al., 2008; Davis, 2007). In this article, we aim to put these findings on a broader international basis by researching the media-related perceptions of political elites in seven advanced democracies. We want to determine how influential politicians perceive the media to be, how they perceive the media’s role in democracy, and whether those perceptions are correlated with their satisfaction with the functioning of the democratic political process itself. The existence of a link between subjective perceptions of the media’s role in democracy and the functioning of democracy could be assumed based on findings about the consequences of ordinary citizens’ media bias perceptions. According to them, low trust in the quality of media coverage undermines citizens’ views of political efficacy and may ultimately reduce trust in the legitimacy of democratic politics (Tsfati and Cohen, 2005). The present paper is interested in whether such a spillover effect can also be observed for political elites who are key players in, and shapers of the democratic process in the democracies studied.

This article addresses this question by re-analysing a survey of politicians of seven advanced European democracies (Austria, Denmark, France, Germany, Spain, Sweden and Switzerland) conducted between 2008 and 2010. The fact that the article uses data gathered some years ago does not diminish the value of the study because *a)* subjective perceptions of policymakers regarding the effects of the media are unexplored but potentially significant for their behaviour, and *b)* the fundamental structures associated with mediatisation still shape contemporary hybrid media systems (Chadwick, 2017). Taking the example of the United Kingdom but also looking at similar situations in other advanced democracies, Chadwick (2017: 56) points out that, ‘despite all of these developments online, older news media organisations continue to play the pivotal roles in [British] politics’. Studies from other European democracies pointing to the undiminished agenda-setting power of the news media (e.g., Djerf-Pierre & Shehata, 2017) underline that Chadwick’s assessment can be generalised to other advanced European democracies.^1^

In the last decade, maybe as a response to perceived media influence on the public agenda, many popular political figures have taken to social media and developed disruptive and sometimes inflammatory discourses. Politicians’ subjective perceptions regarding the influence and performance of the news media may explain their patterns of social media communication.

Although media influence has grown across liberal democracies, our study considers that politicians’ perceptions concerning the media’s political influence and performance as a provider of public information may vary across media systems and political communication cultures (PCCs) (Hallin and Mancini, 2004; Pfetsch, 2014). Even though media systems of the seven democracies studied are relatively similar – they all have a legacy of party-press parallelism, similar cultures of journalism, strong public broadcasting systems and institutional safeguards to ensure autonomy of the media from governmental control (Aalberg et al., 2010; Hallin and Mancini, 2004) – some differences exist: France and Spain have a ‘polarised-pluralist’ media system, whereas Austria, Denmark, Germany, Sweden and Switzerland have a ‘democratic-corporatist’ media system (Hallin and Mancini, 2004; see also Brüggemann et al., 2014). The key difference is that in democratic-corporatist media systems media autonomy from the state is historically more deeply ingrained than in polarised-pluralist systems (Hallin and Mancini, 2004). There also exists a more consensual culture in media systems which reflects a more coalition-oriented political culture.

The PCCs of the countries studied oscillate between three partially overlapping types. While Sweden’s PCC is characterised by strict media autonomy (media-driven type), attempts at strategic influence are more common in Denmark and France (strategic type). Spain shows the strongest advocacy tradition in journalism (traditional type), whereas the (mostly) German-speaking countries, Austria, Germany, and Switzerland, have developed a mix of these types (Pfetsch et al., 2014: 95–96). When analysing politicians’ perceptions, we keep the context differences in mind and empirically test whether they have an impact.

The analysis is motivated by our belief that including the subjective perceptions of concerned individuals allows us to better grasp the political and communication-related implications of mediatisation because ‘mediatization is not a process that just “happens.” (…) It remains one made by humans who give it meaning’ (Hepp, 2020: 10). While the subjective, perceptual side of mediatisation remains understudied, one of the rare studies concludes that the process of mediatisation does not have negative consequences for (Danish) members of parliaments’ (MPs) evaluations of the media over a ten-year period (Elmelund-Præstekær et al., 2011). However, the article covers only one country and is restricted to three specific perceptions that may be context-dependent. The present article focuses on a broader set of perceptions of the media’s role in politics and investigates how they are related to each other and to perceptions of the functioning of democracy in seven democracies. It thus aims to update extant knowledge about politicians’ experiences of mediatisation by showing a broader picture of their subjective media-related perceptions and evaluations.

## Media influence from a systemic perspective

At the macro level, the relationship between media and politics can be grasped through the concept of the mediatisation of politics. This refers to a progressive power shift in this relationship from political actors to the media (Asp, 2014; Kepplinger, 2000, 2002; Mazzoleni and Schulz, 1999; Strömbäck and Esser, 2009) and provides the background of this study. Central to mediatisation theory is the increased dependency of political actors on rules set by the media for access to the mass media public sphere (Asp, 2014; Kepplinger, 2000). As an effect, mediatisation somehow redistributes the roles of media and political actors (Asp, 2014; De Beus, 2011). Media influence over the issues and tone of political coverage has increased since the age of television. According to Kent Asp, we are now in a situation in which the straightforward reporting of events created by political actors has been replaced by ‘interpretive journalism of media defined events’ (2014: 362). Mediatisation also entails a claim by media professionals to serve the public interest better than politicians and a dominance of journalistic voice and narratives in political coverage (Blumler and Gurevitch, 1995). Journalistic voice means that journalists refuse to accept the facts and interpretations supplied by politicians and act as a filter and opponent. They take what politicians provide as raw material and select, frame, and interpret it according to their standards before it flows as political information to an audience. The trend to intervene in politics, rather than following the agenda of political actors, is present in both, democratic-corporatist and polarised-pluralist media systems (Allern and Blach-Ørsten, 2011; Humanes and Roses, 2018; Neveu, 2002) and it can be argued that it gradually redefines politicians’ rules of access to the media agenda. In sum, ‘The mass media have become an omnipresent, highly consequential “environmental factor” that sometimes irritates, interferes, or even obstructs political processes’ (Esser, 2013: 156). In the view of politicians, the media may have progressively turned from enabling agents during the time of the party press into a veto player for political actors.

The structural capacity of the media to define issues and set the tone of public debate corresponds with the motivations of journalists who act on their behalf. Journalists have a strong tendency to produce meaning and transmit values to the public, rather than restrict their activities to objective reporting. (Donsbach and Patterson, 2004; Hallin and Mancini, 2004; Kepplinger, 2009). Although recent journalist surveys show only moderate levels of interventionist role conceptions (Hanitzsch et al., 2016), one has to keep in mind that surveys may underestimate their real importance (Mellado et al., 2017). Studies of journalism have found strong indications that an interventionist profile and critical watchdog role are predominant in political reporting in democratic-corporatist and polarised-pluralist media systems (Esser, 2008; Humanes and Roses, 2018).

The structurally entrenched influence of autonomous media on public information, debate and opinion may raise questions about the political role and influence of the media among policymakers. In modern political theory, the news media’s role is seen as limited to an intermediary between the people and their representatives, not a competing source of political power (e.g., Powell, 2004). Yet, although politicians formally remain decision makers the media reign into their domain. They do so by setting the frames of the coverage and often grant actors who adapt to those frames better access and friendlier coverage (Asp, 2014). By contrast, political actors who refuse to adapt to the media agenda incur the risk of being marginalised or negatively framed (Kepplinger, 2000: 157–163).

Although scholars like Wolfsfeld (2013) nuance the claim that media could take a dominant position vis-à-vis political actors by positing that political action would precede the media’s response to events, he acknowledges that the media have the power to transform political events by giving disproportionate attention to specific aspects or by promoting specific frames.

In summary, mediatisation is likely not experienced as a politically neutral process by politicians. Rather, we assume that, under the condition of advanced mediatisation, leading media outlets are perceived as competing agenda setters in the political process and as problematic mediators. If politicians perceive the media’s role in politics as excessive, this may even reduce their perception that they can fulfil their own roles – in other words, articulating political standpoints independently of the media agenda and showing responsiveness to their voters – which in turn may undermine their satisfaction with the political system. To uncover the potential transformation of the power relation between media and politics and how it affects the political system, it is promising to leverage the subjective views of politicians as an empirical yardstick.

## Perceptions of the media’s role and influence

Politicians’ subjective perceptions have moved to the research agenda in the past 10 to 15 years. Several survey-based studies have shown that politicians from many countries perceive media influence as strong and negative (Kepplinger, 2009; Soontjens et al., 2020; Strömbäck, 2011; Van Aelst et al., 2008; Vesa et al., 2015; Walgrave, 2008). In particular, politicians perceive the media as dominant political agenda setters and believe that they control the careers of politicians (Maurer, 2011; Lengauer et al., 2014; Van Aelst et al., 2008). These perceptions are remarkably homogenous across several advanced European democracies with democratic-corporatist media systems. Surveyed politicians tend to believe that many journalists act out of self-interest in political coverage and put the goals of their media organisations before the public interest (Brants et al., 2010; Kepplinger, 2009). Politicians also tend to associate strong media influence with an inclination of journalists to promote their own political views: MPs believe that journalists are obsessed with the desire to exercise political power (Brants et al., 2010; Van Aelst et al., 2008; Walgrave, 2008). Håkansson and Mayerhöffer (2014) found that the perception that journalists see it as important to give their own opinions on political matters lowers politicians’ confidence that the media strengthen the link between citizens and democracy.

How politicians evaluate the performance of the media beyond evaluating journalists is, unfortunately, seldom assessed. The only study to our knowledge that has undertaken such an evaluation is Elmelund-Præstekær et al.’s (2011) longitudinal research on the effects of mediatisation in Denmark. The conclusion for the long-term perspective is that ‘mediatization has not made the MPs more critical toward the media’s role in politics in general’, and the authors go on to claim that ‘judging by the view held by national politicians, the role of the media in modern democratic politics may not be as problematic as (…) some scholars would like us to think’ (p. 392). While this conclusion may be warranted for the evolution of the measured attitudes over the examined period, it cannot be taken for a general evaluation of the media’s performance due to a narrow measurement and the fact that the context is not representative of other democracies.

There is still a scarcity of more comprehensive research on the correlates of media influence perceptions, especially since their relevance for politicians’ behaviour has already been shown (Cohen et al., 2008). Cohen et al. (2008) found that politicians’ beliefs that the media influence the public strengthen their motivations and efforts to be covered by the media. Influence perceptions also affect political attitudes, as shown by Dohle et al. (2012) who found that a negative evaluation of media influence among MPs leads to a positive attitude towards curbing that influence.

The existence of a link between attitudes (such as trust) towards the media and the political system has been established for the general population. Tsfati and Cohen (2005) suggest that among average individuals, there is a correlation between trust in news and trust in the functioning of democracy. They argue that perceptions that others are badly informed about political matters decrease trust in the fairness and democratic quality of political decision making. The authors conclude that ‘in situations of high involvement, trust in news media is an especially crucial component of trust in democracy’ (Tsfati and Cohen, 2005: 33; see also Ariely, 2015). Further research suggests that the strong association between trust in the media and trust in democracy among a population can be generalised across democratic societies (Tsfati and Ariely, 2014). It is not unlikely that a similar association exists for political elites.

### Hypotheses

**Figure 1.**
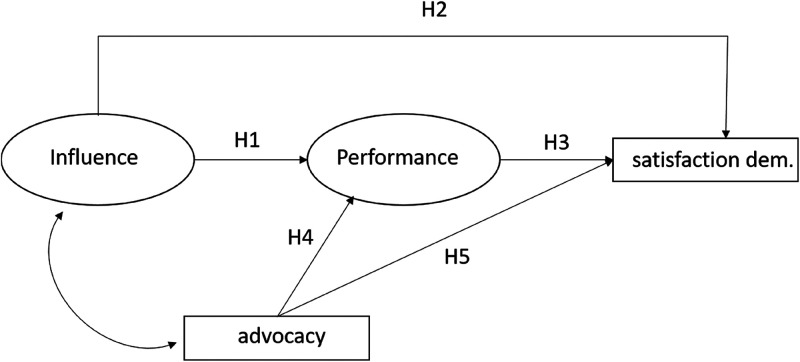
Theoretical structural equation model. Effects of politicians’ function, age and gender (controls), and error labels are omitted for clarity reasons.

Despite great agreement that mediatisation has induced a power shift between the media and the core actors in political systems, it remains unclear whether politicians subjectively perceive media influence as functional or dysfunctional in the democratic process. Based on the arguments and results presented, we propose a set of hypotheses predicting the interplay of some of their media- and democracy-related perceptions and evaluations (Figure 1). In detail, the model contains five hypotheses about the consequences of politicians’ perceptions of media influence, media performance, and journalists’ role performance. Since perceptual studies of politicians (Brants et al., 2010; Van Aelst et al., 2008) have established that perceived media influence makes politicians doubt the performance of the media with respect to their role as public informant, our first hypothesis (H1) reads:

H1: The more influential politicians perceive the media to be as informal decision makers in the political process, the more problematic they will perceive the news media's performance in regard to conveying information to the public.

If the media take over the role of political agenda setting and career making from parties in the political process, this could be costly for politicians in several ways. First, adaptation entails the danger of losing contact with constituencies whose demands are under-represented in the media. Second, politicians have little influence over the issues and frames the media play up in election campaigns, which makes the success of their own media communication uncertain. Thus, politicians may develop feelings of frustration and disempowerment regarding their role in the political communication process as a consequence of strong media influence (Maurer & Arendt, 2016). This may, in turn, reduce their satisfaction with the functioning of the political system. Hence, our second hypothesis (H2):

H2: The more influential politicians perceive the media to be in the political process, the less satisfied they will be with how democracy functions.

We assume furthermore an effect running from politicians’ perceptions of the performance of the media as public information providers on their satisfaction with the functioning of democracy, similar to the effect found for citizens (Tsfati and Cohen, 2005):

H3: Less positive perceptions of the news media's performance in regard to public information will reduce politicians’ satisfaction with how democracy functions.

Finally, from Brants et al.’s (2010) results can be inferred that the more politicians perceive journalists to act as political advocates, the more they will develop a negative perception of the performance of the media as an information channel. Behind this may be hostile media perceptions to which political elites fall prey (Matthes et al., 2019). Given the crucial gatekeeper role of journalists, we assume that politicians’ beliefs that journalists perform an advocacy role will reduce their satisfaction with the functioning of democracy due to fears that content will be biased against their positions. This results in the fourth and fifth hypotheses:

H4: The more politicians perceive journalists as political advocates (rather than neutral information providers), the more problematic they will perceive the performance of the news media in regard to public information.

H5: The more politicians perceive journalists as political advocates, the less satisfied they will be with the functioning of democracy.

## Materials and Methods

### Respondents

The study focuses on politicians from seven mature democracies with crucial commonalities in their systems of government and media: Austria, Denmark, France, Germany, Spain, Sweden and Switzerland. Data were collected as part of the ESF-funded research project Political Communication Cultures in Europe (cf. Pfetsch, 2014). The population of eligible politicians was defined as members of national legislatures or governments (including top-level advisers). The rationale for this was that respondents should have an election mandate (or at least depend on elections in the case of top-level political advisers). The population was divided into two sub-groups: MPs, preferably in a leadership role, and government officials. In France, a few city mayors replaced MPs due to the high refusal rate in this group.

The sampling followed a positional approach, meaning that individuals in leadership roles in either a parliament (such as presidents, chairpersons, speakers and deputies of the house, a party group, or a committee) or a government (chancellor, minsters, secretaries of state, and top-level advisers to a minister in the case of France) were first identified and approached. The targeted sample size was set to N = 100 per country, to ensure that each country has about the same weight in the total sample. It was permitted to replace holders of leadership positions who declined the invitation to participate with MPs without a leadership role (i.e., backbenchers or junior MPs). The positional approach enhanced comparability of respondents between countries, although the national investigators who were entrusted with the selection of positions were given some flexibility with respect to necessary replacements for refusals or dropouts. The different relations of MPs to government officials in the samples across countries reflect the fact that in some parliaments, more leadership roles exist than in others or are the result of high refusal rates in one group. In the case of France, as MPs were virtually impossible to reach due to an ongoing regional election campaign at the time of the fieldwork, more government officials were included. The surveys were fielded by principal investigators in spring 2008 (November 2009 in France). In Spain and Austria, national snap elections interfered with data collection in 2008, which had a negative impact on the response rate.

There were a few country-specific particularities. In Austria, a few MPs of the European Parliament were added as replacements for busy national-level MPs; the Swiss and Danish governments were completely left out because ministers were judged unavailable for scientific investigations by the principal investigators; and in Germany, MPs with high leadership functions systematically refused to participate, leading to participant MPs not being in leadership positions. Despite the foreseeable high refusal rate of this elite survey, the achieved sample allows for testing the hypothesised relationships because it contains a sufficient number of political elites from the legislative and executive branches from all countries. However, in no country is the sample representative of the entire political elite (Table 1).

**Table 1.** Participants and response rate by institution and country.

	Country						
	*Austria*	*Denmark^a^*	*France^b^*	*Germany*	*Spain*	*Sweden*	*Switzerland^c^*
Respondents MPs (n)	27	27	7	49	18	75	65
Response rate (%)	23.5	58.6	–	21.1	28.6	64.6	34.4
Respondents Gov't (n)	7	0	38	6	14	12	0
Response rate (%)	24.4	0.0	42.0	26.7	30.6	22.4	–
Sample (n = 345)	34	27	45	55	32	87	65

*Note:* In Austria, Denmark, Sweden and Switzerland a fourth category, interest group elites, are included in the calculation of the overall response rates, but those respondents were excluded from the analyses.

^a^ No government elites responded.

^b^ Due to a loss of information about the number of contacted respondents no response rate could be calculated.

^c^ No government elites were sampled.

Of the total number of respondents for whom we have full information (*n* = 345), the majority were MPs (*n* = 268; 78%), and the rest held high offices in national governments or in rare cases as mayors (*n* = 77; 22%). Of the participants, 31.3% were female, and they had about 15 years of average work experience as politicians. The majority (35.9%) were between 50 and 59 years old, followed by the 40 to 49 age group (23.5%). The standardised interviews, which lasted about 30 min on average, were conducted by telephone, as face-to-face interviews, or in some cases with a questionnaire sent by mail. Face-to-face and telephone interviews were conducted by trained interviewers of commissioned survey institutes or by members of the research teams.

### Measures

The questionnaire contained, inter alia, measures for the constructs discussed and socio-demographic controls. The constructs were measured using one or several items. Perceptions of media influence on the political agenda and careers (*Influence*) and the perception of the media’s performance in regard to public information (*Performance*) were designed as latent variables measured with multiple items, whereas politicians’ perceptions that journalists are political advocates and their satisfaction with the functioning of democracy were measured as observed variables with one item each. All items had five-point Likert-type answer scales with labelled poles. To gauge politicians’ satisfaction with the functioning of democracy, a standard indicator that stems from political culture research was used (‘In general, how satisfied are you with the way democracy works in this country?’ 1 = ‘very dissatisfied’ to 5 = ‘very satisfied’; *M* = 3.55; *SD* = .90).

The perception of the media’s performance as an information channel was modelled as a latent variable measured by three indicators. The indicators tapped different but related aspects of that role: (i) the perceived performance of the news media (‘How would you say the media are informing citizens on political matters?’ 1 = ‘very bad’ to 5 = ‘very good’; *M* = 2.99; *SD* = .83), (ii) the perceived effect of media coverage on citizens’ political trust (‘Do you think that the current media coverage contributes to an increase or a decrease in political trust?’ 1 = ‘leads to a decrease’ to 5 = ‘leads to an increase’; *M* = 2.29; *SD* = .77), and (iii) the media’s perceived overall impact on the functioning of democracy (‘Now I would like to have your opinion on the media’s impact on how well democracy functions in this country’; 1 = ‘very negative’ to 5 = ‘very positive’; *M* = 3.12; *SD* = .88). The resulting averaged scale for *Performance* had a Cronbach’s *α* of. 63.

*Influence* was also measured as a latent variable by combining two indicators, tapping the media’s influence on the policy agenda (‘The media decide which issues are important in politics while politicians have little impact on this matter’; *M* = 3.34; *SD* = .97) and their career-controlling power (‘Mass media make and break politicians’; *M* = 3.45; *SD* = 1.01; Cronbach’s *α* for scale = .55; *r* = .38; *p* < .001). Answer scales for both items ranged from 1 = ‘strongly disagree’ to 5 = ‘strongly agree’. Factor loadings for both indicators on *Influence* were set to equality in the analysis.

Finally, the extent to which politicians view journalists in an advocacy role was measured as an observed variable with the item ‘Journalists cover political issues to voice particular views on political developments’ (1 = ‘strongly disagree’ to 5 = ‘strongly agree’; *M* = 3.11; *SD* = .95).

To avoid underspecified regression models, we included a number of important controls. Besides age, work experience as a politician, and gender, we controlled on all endogenous variables the effects of politicians’ office and whether they belonged to the governing party (coalition) or the opposition. We used structural equation modelling with the ‘R lavaan’ package (Rosseel, 2012) to test the hypothesised model. The comparative fit index *(CFI)*, root mean square error of approximation *(RMSEA)*, and standardised root mean square residual *(SRMR)* served as criteria to evaluate the model fit. If not mentioned otherwise, we report unstandardised path coefficients (*b*).

## Results

**Figure 2.**
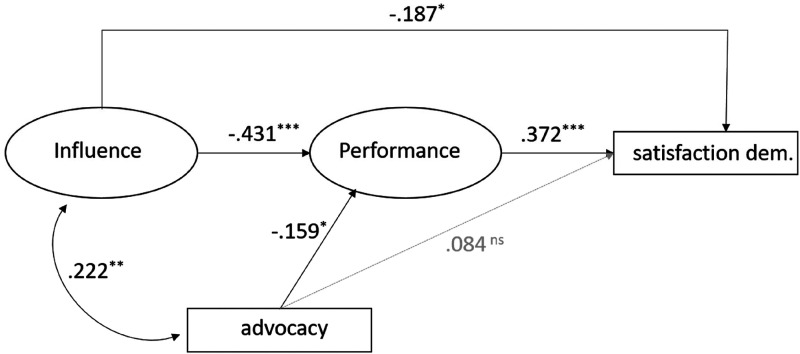
Effects of politicians’ perceptions regarding (a) journalists’ role, (b) media influence on their perception of media performance and their satisfaction with democracy. Data from seven Western European democracies. Black arrows stand for influence, grey dashed arrows stand for insignificant paths. Entries are standardised path coefficients. Model goodness of fit: χ2 = 15.415, df = 11, *p* = .164, CFI = 0.98, TLI = 0.97, RMSEA = 0.034, SRMR = 0.030. Explained variance of criterion variables: Media Performance *R^2^* = 34.4%; Satisfaction with Democracy *R^2^* = 63.2%.

The empirical relationships between the variables of our hypothetical model were tested in a structural equation model (see Table 2 and Figure 2). The fit of the model was excellent (*χ2* = 15.415; *df* = 11; *p* = .164; *CFI* = 0.95; *TLI* = 97; *RMSEA* = 0.034; *SRMR* = 030). As predicted in hypotheses one and two, there was a negative effect of perceived media influence on *Performance* (*b* = −.470; *p* < .001) and on satisfaction with the functioning of democracy (*b* = -.237; *p* < .05). Our third and fourth hypotheses predicted that politicians’ perceptions that journalists primarily act as political advocates have a negative effect on how politicians evaluate the media’s democratic role performance and how satisfied they are with the actual functioning of the democratic process. Yet, we found only an effect on the evaluation of the media’s role performance (*b* = -.113; *p* < .05). That is, the more politicians perceive journalists as political advocates, the less functional they will evaluate the mass media’s political role.

**Table 2.** Unstandardised structural equation modelling path coefficients.

	Media Performance	Satisfaction with Democracy	Media Performance	Satisfaction with Democracy
Predictors	b (SE)	b (SE)	b (SE)	b (SE)
Journalists Perceived as Advocates	−.113 (.047)*	.080 (.051)	−.081 (.052)	.060 (.054)
Perceived Media Influence	−.470 (.104)***	−.273 (.124)*	−.558 (.124)***	−.298 (.153)^(*)^
Eval. of Media Performance	−	.497 (.112)***	−	.449 (.131)***
*Controls:*				
Age			−.008 (.005)	.006 (.005)
MP ( = 1) vs. Gov			.004 (.054)	−.086 (.058)
Gender (1 = female)			−.166 (.102)	.031 (.109)
Explained Variance (*R^2^*)	.24	.22	.31	.22

*Note.* ***p < .001; **p < .01; *p < .05; (*) *p* = .052.

We also predicted that *Performance* influences satisfaction with democracy. When the role of the public’s main provider of political information is deemed harmful by politicians, this should reduce their trust in the functioning of democracy. Indeed, we found a strong effect in the expected direction (*b* = .497; *p* < .001), which outweighs the total effect of perceived media influence (*b* = -.506; *p* < .001) on satisfaction with democracy. The total effect goes beyond the direct effect of *Influence* since we also found an indirect effect (*b* = -.160; *p* < .001) through *Performance*. Significance levels for the indirect effects were calculated using R lavaan’s implemented delta method (Sobel, 1982).

The perception that journalists behave like political advocates had a small indirect effect on satisfaction with democracy through *Performance* (*b* = -.056; *p* < .05) but no direct effect. We can draw the conclusion that *Performance* is a strong direct predictor of politicians’ satisfaction with the state of democracy and also acts as a mediator.

The strong effect of *Performance* on satisfaction with democracy suggests that the political role of the media is deeply entrenched in politicians’ thinking. When controls for politicians’ age, gender, and sector (legislature versus government) were added to the regression models, the significance of the path coefficients associated with the direct effect of *Influence* and the indirect effect of perceived journalistic advocacy on satisfaction with democracy disappeared, but no other relationships changed (Table 2). Nevertheless, the effect of *Influence* on the outcome was still marginally significant. None of the control variables had a significant direct effect on *Performance* and satisfaction with democracy.

Finally, the question of whether the effects are uniform between the countries studied was tested. In the pooled sample, we have respondents from five countries with democratic-corporatist media systems and from two countries with polarised-pluralist media systems. An inspection of the main path coefficients per country (Table A1, Appendix) shows that the effects are all in line with those of the pooled model, with the only exception that the effect of *Performance* on satisfaction with democracy in Austria is negative. We conducted a multi-group *Structural Equation Model (SEM)* analysis with R lavaan, formally comparing an unrestrained model (all coefficients are free to differ across countries) to a model in which the effects of *Influence* on *Performance* and *Performance* on satisfaction with democracy were constrained to be equal for all countries. The results show that the constrained model had no worse fit than the unrestrained model (*χ2* (diff.) = 11.028; *df* (diff.) = 12; *p* > .05; chisq. = .526), suggesting that the effects revealed in the pooled model can be assumed to hold for all countries. This robustness check formally confirms the hypotheses according to which there is a negative effect of perceived media influence on perceptions of the media’s role and the functioning of democracy across European democracies regardless of media system differences.

The strongest path in the model runs from politicians’ perceptions that the media exert a strong influence on political matters through scepticism about the quality of the information provided by the media (Performance) to (dis)satisfaction with the actual functioning of democracy. It is worth highlighting that the structural model as a whole fits the data well, supporting the hypothesised relationships. Moreover, the negative effect of perceived media influence on perceptions of the media’s performance as a provider of information is in line with the findings of related research (Brants et al., 2010; Van Aelst et al., 2008).

## Discussion

Politicians’ subjective views about the media revealed in this cross-national survey-based study contradict the conclusion about an unproblematic reaction of politicians to media influence (Elmelund-Præstekær et al., 2011) in important respects. The results show that media influence perceptions, perceptions of ill-informed citizens due to poor media performance, and perceptions of poor functioning political institutions go hand in hand. The fact that there were no strong country differences suggests that the pattern of perceptions found is typical for politicians in advanced, media-driven democracies. For them, political-democratic processes are negatively affected when they perceive the media gaining strong influence over political agendas and leadership. The effect of media-related perceptions – especially concerning the media's performance as public informants – on democracy-related feelings among politicians mirrors the same relationship among citizens (Ariely, 2015). Together, these findings corroborate the sensitivity of the democratic political process to media influence. Hostile media perceptions may contribute to this effect, as political elites, just like ordinary news users, tend to perceive political coverage as impactful and biased against their views (Matthes et al., 2019).

The pattern of subjective views found can be taken as a sign of a transformed relationship between the media and a significant portion of the political elite in postmodern democracy. The antagonistic relationship between some political parties that garner large vote shares and large parts of the ‘mainstream’ media epitomises this change. On the other hand, the surprisingly strong readiness to adapt to media agenda-setting and framing by other political actors is also indicative of the transformation in power relations.

When compared with theoretical accounts of the media-politics relationship, the structure of perceptions in principle corresponds to the power distribution between politicians and the media in models of advanced mediatisation, audience, and post-democracy (De Beus, 2011; Crouch, 2004). Audience democracy depicts a situation in which the media sketches out a path through their agenda-setting power, and politicians’ role is limited to executing this agenda. In this optic, the findings suggest that politicians are sensitive to the legitimisation and accountability issues that arise in a post-democratic constellation in which the media push politicians to adopt a media-induced political agenda.

On the micro-level, the negative effect of media influence perceptions on media performance evaluations suggests that mediatisation means more for many political actors than adapting to a changing presentational style or a faster rhythm of publication. In their view, this process in which the media gain power over the themes of public debate seems to be associated with risks for democracy due to insufficient quality of public information.

The results of this study can serve as an explanation for politicians’ efforts to become their own media. The correlated perceptions of strong media influence and poor quality of information may have fostered the adoption of social media handles by politicians. Especially politicians of populist parties use their handles to delegitimise the media in different ways (Van Dalen, 2021). These attacks may undermine media trust among many who follow politicians on social media. Another reaction to negative views of the legacy media among politicians is the attempt to create or support new media organisations as an alternative with the risk to exacerbate political polarisation and audience fragmentation.^2^

Replacing communication through the news media by using social media can be a strategy dissatisfied politicians adopt. Yet, it means that part of the political debate takes place increasingly outside journalistic media, at the risk that it becomes less structured, more emotional, and more prone to polarisation of opinions. Many politicians, other political activists, and (some) citizens took to social media in the 2010s, which created a polarised, shrill, and transgressive debate outside traditional news media. As journalists also follow this new forum for public debate, some polarised opinions popping up there spill back over to journalistic content. Through social media, political outsiders not in line with the media agenda, such as Donald Trump or Jean-Luc Mélenchon, could gain enough media attention to be nominated as presidential candidates (Wells et al., 2016). Likewise, in European democracies, it is often the opponents of established parties whose communication relies most on social media where the tone of the messages tends to be more ‘populist’ than in the news media with potential effects on followers (Ernst et al., 2019; Spieß et al., 2020).

## Conclusion

This investigation has revealed implications of the process of perceived mediatisation among politicians in modern democracies with autonomous media organisations. As the findings have shown, perceived media influence is clearly linked with doubts about whether the media still serve democracy well and even doubts about the functioning of political institutions. This underlines that it is important to consider a broader picture of perceptions and evaluations than previous studies that have analysed media influence perceptions of policymakers in isolation of other political perceptions. The present study has pointed to the consequences and ramifications of these perceptions, affecting especially views of media performance and political institutions. We could also establish the generalizability of the perceptual relationships found across a range of advanced democracies.

Finally, our results provide a hint about the perceptions that could encourage politicians to avoid news media and seek other channels to reach the public than journalistic news media. Such channels are, like social media platforms, often used to attack journalists and the news media as an institution, which can trigger and reinforce existing resentment in the wider public. Considering the results of this study, we may interpret this trend as a way adopted by some politicians to break the media dominance in politics and counter perceived deficiencies of the media in regard to their information function.

Of course, our study has limitations. It would have been desirable to measure politicians’ perceptions of journalistic roles, media influence, and media performance with additional items to cover these concepts more effectively. That said, we are satisfied that we had multiple item measures and did not have to rely on single items. There is doubt about causality in the interrelations of perceptions. Although we ran different models representing different causal paths, cross-sectional data are not ideally suited for investigating the interplay of perceptions. Nevertheless, the fact that the observed effects are in line with preceding studies, as well as justified by theoretical considerations, makes us confident concerning their robustness. While a task for future research is to apply a panel design to achieve higher confidence about the direction of influence in the relationships, this is a great challenge for the type of population, particularly in cross-national research. Last not least, it would be useful to include perceptions about new media and social media platforms in a further study.

## Notes

1. As Djerff-Pierre and Shehata put it: ‘Our findings clearly suggest that public opinion is no less responsive to the media agenda in today's high-choice media environment than during the low-choice era (…) particularly when considering the collective agenda of several prominent news media outlets’. (2017: 751).

2. For example, the rightwing populist German opposition party AfD, which is critical of the legacy media, has announced in July 2022 that it will consider to found or support (online) media as a response to the consistently poor treatment by most legacy media (RND, 2022).

## Appendix

[Table table3-17480485221146088]

**Table A1. table3-17480485221146088:** Unstandardised path coefficients across countries (appendix).

	Influence → Performance	Influence → Satisfaction	Performance → Satisfaction
Countries	b (SE)	b (SE)	b (SE)
Austria (n = 34)	−.194 (.190)	−.912 (.639)	−.891 (2.108)
Denmark (n = 27)	−.868 (.401)	−.010 (.619)	.572 (.507)
Germany (n = 55)	−.457 (.280)	−.510 (.315)	.364 (.192)*
Spain (n = 32)	−.585 (.381)	−.652 (.828)	.180 (.784)
Sweden (n = 87)	−.552 (.160)	−.552 (.160)	.210 (.255)
Switzerl‘d (n = 65)	−.183 (.181)	−.137 (.222)	.803 (.284^)**^
France (n = 45)	−.667 (2.519)	−2740 (8975)	.718 (1.134)

*Note.* ***p* < .01; **p* = .058.
